# Association between the non-high-density lipoprotein cholesterol to high-density lipoprotein cholesterol ratio and sarcopenia: evidence from CHARLS

**DOI:** 10.3389/fpubh.2025.1585986

**Published:** 2025-04-30

**Authors:** Changbo Sun, Honggang Jiang, Hongmei Zhu, Zhen Luo, Shiyi Wang

**Affiliations:** Department of Gastroenterology, Ningbo Hospital of Traditional Chinese Medicine, Affiliated With Zhejiang Chinese Medical University, Ningbo, China

**Keywords:** NHHR, sarcopenia, lipid metabolism, dyslipidemia, Chinese population

## Abstract

**Background:**

Recent studies have highlighted an association between lipid disorders and sarcopenia. The role of the non-high-density lipoprotein cholesterol to high-density lipoprotein cholesterol ratio (NHHR) has not been explored among Chinese adults. This study aimed to investigate the association between the NHHR and incident sarcopenia in the Chinese population.

**Methods:**

The study included a total of 4,046 participants aged 50 years and older without a history of sarcopenia, from the China Health and Retirement Longitudinal Study (CHARLS). A multivariate logistic regression model and a restricted cubic spline model were used to investigate the association between NHHR and sarcopenia. Subgroup and sensitivity analyses were conducted to assess the robustness of the findings.

**Results:**

A total of 309 (7.6%) participants were newly diagnosed with sarcopenia in the 2015 wave. Participants in the highest NHHR quartile (≥3.99) had a significantly lower adjusted odds ratio for sarcopenia (OR = 0.40; 95% CI, 0.280.58; *p* < 0.001) compared with those in the lowest quartile (<2.24). Restricted cubic spline analysis revealed a nonlinear relationship between NHHR and sarcopenia risk (p for nonlinearity <0.05). In piecewise regression models, the adjusted OR for sarcopenia was 0.65 (95% CI, 0.550.78, *p* < 0.001) among participants with NHHR <4.4, whereas no significant correlation was observed among those with NHHR ≥ 4.4. No significant interactions were found between NHHR and age, sex, hypertension, or diabetes in stratified analysis (p for interaction >0.05).

**Conclusion:**

There is an inverse relationship between NHHR and sarcopenia risk in the Chinese population. A higher NHHR is associated with a lower risk of sarcopenia below the inflection point, beyond which NHHR is no longer significantly associated with sarcopenia risk.

## 1 Introduction

Sarcopenia is a condition characterized by an age-related loss of muscle strength, which is often paired with a decrease in skeletal muscle mass and/or impaired physical performance (1). The condition is strongly linked to adverse outcomes, including diminished physical function, increased risk of falls, metabolic problems, cognitive decline, and higher mortality rates (2–4). Globally, sarcopenia affects 5–10% of the general population and 10–27% of individuals aged 60 years and above (5, 6). In China, the prevalence of sarcopenia among older adults ranges from 11 to 18% (7).Sarcopenia is an emerging public health concern, due to rising healthcare costs worldwide.

The non-high-density lipoprotein cholesterol to high-density lipoprotein cholesterol ratio (NHHR) is an emerging lipid marker with significant potential for predicting the risk of various diseases, including coronary artery disease, depression, diabetes, and obstructive sleep apnea (8–11). There is evidence suggesting that NHHR is superior to traditional lipid markers in assessing the severity of atherosclerosis in middle-aged and older individuals in China (12). In addition, NHHR has been linked to both all-cause and cardiovascular-related mortality in individuals with diabetes or prediabetes (13).

Several studies have investigated the relationships between common lipoproteins, such as the TG/HDL-C ratio (14) and HDL-C (15, 16), and sarcopenia. Recent research has revealed a positive association between NHHR and sarcopenia in American adults (17–19). However, studies examining this relationship in other populations are limited. From the China Health and Retirement Longitudinal Study (CHARLS), we collected data on sarcopenia, NHHR, and related factors. We then analyzed the incidence of sarcopenia via the 2015 wave of data and performed a stratified analysis.

## 2 Methods

### 2.1 Study population

CHARLS is a large prospective cohort study that provides high-quality microdata in China, with a response rate of 80.5%. The baseline survey was conducted in 2011 and covered 28 provinces (autonomous regions and municipalities) nationwide. A multistage sampling method was used, with probability proportional sampling applied at both the county and village levels. The health status of the participants was followed up every 2 to3 years. More comprehensive information about CHARLS has been reported elsewhere (20). The Biomedical Ethics Committee at Peking University approved the trial, and written consent was obtained from all participants.

**Figure 1:**
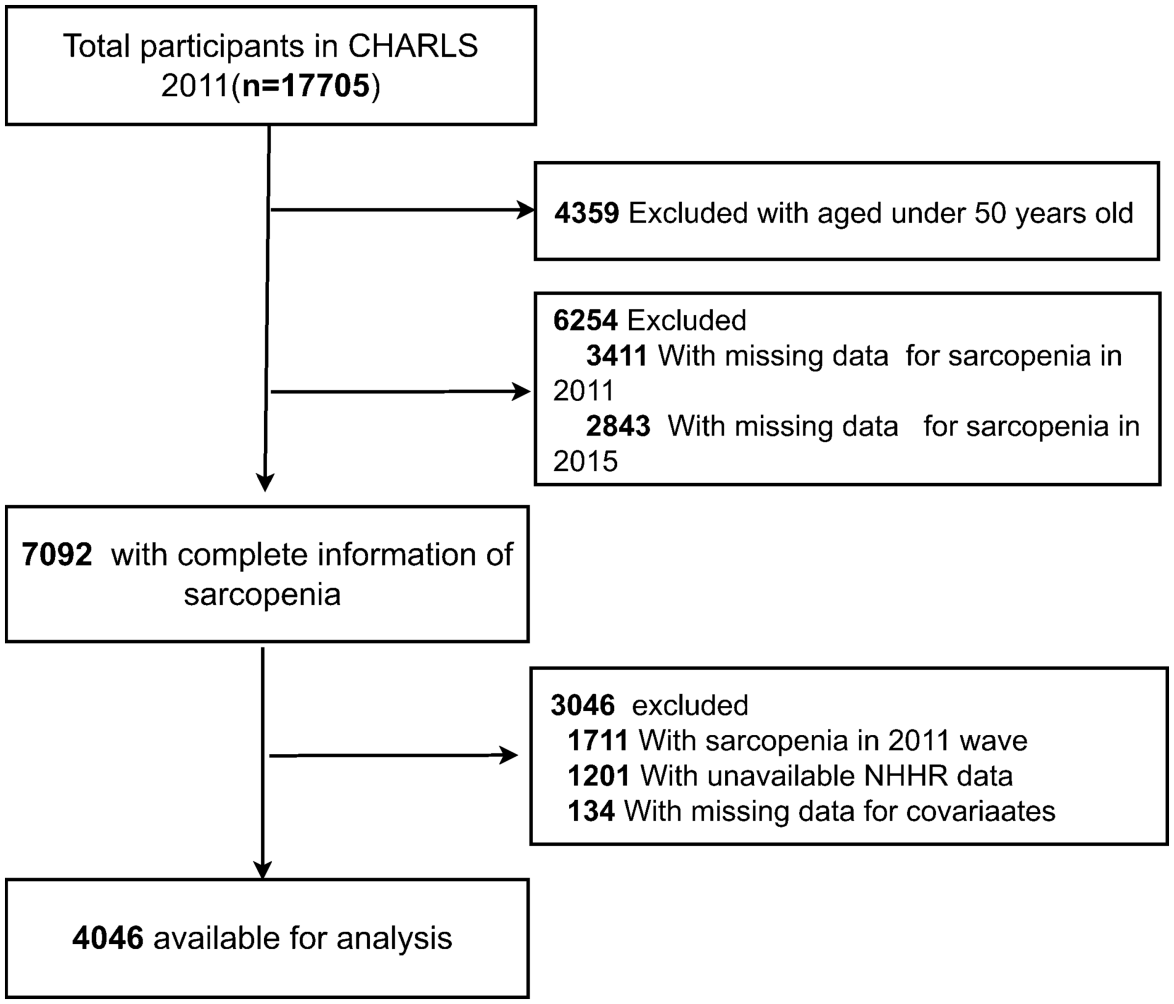
Study flow chart.

In total, 17,705 participants were recruited in the CHARLS 2011 wave. A total of 13,659 participants were excluded for the following reasons: (1) age <50 years in 2011 (*n* = 4,359); (2) missing data on sarcopenia in 2011 (*n* = 3,411), and 2015 (*n* = 2,843); (3) presence of sarcopenia in 2011 (*n* = 1711); (4) missing data on NHHR (*n* = 1,201); and (5) lack of data on other covariates (*n* = 134). Finally, 4,046 participants were classified into the nonsarcopenia group and the sarcopenia group based on their sarcopenia diagnosis in 2015. A detailed flowchart is presented in Figure 1.

### 2.2 Measurements

#### 2.2.1 Diagnosis of sarcopenia

On the basis of the Asian Working Group for Sarcopenia (AWGS) 2019, sarcopenia was defined through muscle strength, muscle mass, and physical performance (1). Muscle strength was measured via handgrip strength. It was considered reduced if handgrip strength was <28 kg for men or <18 kg for women. CHARLS did not measure muscle mass in the participants. Appendicular skeletal muscle mass (ASM) was calculated by a validated anthropometric equation in the Chinese population, which has shown a high agreement with dual-energy X-ray absorptiometry (DXA) measured ASM, and has been widely used in large-scale epidemiological studies (21–23), as follows:


$$\begin{array}{l} ASM=0.193\times \mathrm{weight}\;\left(\mathrm{kg}\right)+0.107\times \mathrm{height}\;\left(\mathrm{cm}\right)\\ \;-4.157\times \mathrm{sex}-0.037\times \mathrm{age}-2.631 \end{array}$$

Sex was set to 1 for males and 0 for females. For muscle mass assessment, ASM was adjusted for height (ASM/Ht^2^) where the lowest 20% ASM/Ht^2^ was the cut-ff value for low muscle mass (22). Accordingly, individuals with ASM/Ht^2^ values <5.69 kg/m^2^ for women and <6.88 kg/m^2^ for men were classified as having low muscle mass.

Physical performance was considered impaired if the walking speed was < 1 m/s, the time to complete the five-time chair stand test was >12 s, or if the participant was unable to complete the test. Participants with low muscle mass and low muscle strength or impaired physical performance were classified as having sarcopenia.

#### 2.2.2 Assessment of NHHR

NHHR was calculated as the ratio of non-HDL-C (mmol/L) to HDL-C (mmol/L), where non-HDL-C was determined by subtracting HDL-C (mmol/L) from total cholesterol (TC, mmol/L) (24).

#### 2.2.3 Assessment of covariates

The covariates used in this study included age, sex, education level, marital status, location of residence, smoking status, drinking status, and chronic diseases (hypertension, diabetes, heart disease, and kidney disease). The covariates were defined in line with a previous study (25).

### 2.3 Statistical analysis

Continuous variables were expressed as the mean values and standard deviations (SDs), and categorical variables were expressed as percentages. One-way analysis of variance, Kruskal–Wallis test, and chi-square test were used to analyze the baseline characteristics and incidence of sarcopenia. Logistic regression analysis was employed to calculate the odds ratios (ORs) with 95% confidence intervals (CIs) for the association between NHHR and sarcopenia.

In addition, restricted cubic spline (RCS) regression with three knots was conducted to assess the potential nonlinear relationship and to explore the dose–response curve between NHHR and sarcopenia. A two–piece logistic regression model with smoothing was used to investigate the threshold between NHHR and sarcopenia. The inflection points were identified by the likelihood ratio test and the bootstrap resampling method.

Interaction and subgroup analyses were performed to investigate the possible influence of sex, age (50–65 vs. ≥65 years), hypertension, and diabetes on the association between NHHR and sarcopenia. We used multivariate logistic regression to investigate the heterogeneity between subgroups.

To address potential confounding factors, we employed propensity score matching (PSM) to minimize the influence of sex differences between the sarcopenia and non-sarcopenia groups. The PSM analysis was performed using a logistic regression model, where sex was the covariate to estimate the propensity scores.

Matching was conducted using a 1:1 nearest–neighbor matching algorithm without replacement, with a caliper of 0.2 to minimize bias. To assess the balance between the matched groups, standardized mean differences (SMDs) were calculated, and a threshold of SMD < 0.1 was considered an acceptable threshold.

In addition to sex-based PSM, we performed multivariate logistic regression analyses to adjust for other potential confounders, including age and smoking habits. The robustness of the non-linear association between NHHR and sarcopenia was evaluated through unmatched data.

The analyses were carried out using R Statistical Software (Version 4.4.2, http://www.R-project.org, The R Foundation) and the Free Statistics Analysis Platform (Version 2.1, Beijing, China). Two-sided values of *p* < 0.05 were considered significant.

## 3 Results

### 3.1 Baseline characteristics

Table 1 presents the baseline characteristics of the 4,046 participants in this study, 309 of whom developed sarcopenia. The mean age was 60.4 (6.9) years, and 1732 participants were female. Compared with those without sarcopenia, the individuals with sarcopenia were more likely to be older, and female, have lower levels of education, be unmarried, live in nonrural areas, have higher HDL-C levels, and have lower NHHR values. Before matching, the sarcopenia and non-sarcopenia groups differed in sex, age, and smoking habits. After sex-based PSM, the SMDs for all covariates were below 0.1, indicating an adequate balance between the groups (Supplementary Table S1).

**Table 1:** Characteristics of the participants.

Characteristic	Total (*n* = 4,046)	Non-sarcopenia (*n* = 3,737)	Sarcopenia (*n* = 309)	*p* value
Age (SD)	60.4 (6.9)	60.0 (6.7)	65.0(7.0)	<0.001
Sex				<0.001
Female	1732 (42.8)	1,561 (41.8)	171 (55.3)	
Male	2,314 (57.2)	2,176 (58.2)	138 (44.7)	
Education				<0.001
No formal education	1937 (47.9)	1740(46.6)	197 (63.8)	
Primary school	1,001 (24.7)	933 (25.0)	68 (22.0)	
Middle school	740 (18.3)	709 (19.0)	31 (10.0)	
High school or above	368 (9.1)	355 (9.5)	13 (4.2)	
Married	3,637 (89.9)	3,382 (90.5)	255 (82.5)	<0.001
Location in rural	1,389 (34.3)	1,316 (35.2)	72 (23.6)	<0.001
Current smoker	1850 (45.7)	1732 (46.3)	118 (38.2)	<0.001
Current drinker	1788 (44.2)	1,669 (44.7)	119 (38.5)	0.042
Hypertension				0.211
No	2,160 (53.4)	1984 (53.1)	176 (57.0)	
Yes	1886 (46.6)	1753 (46.9)	133 (43.0)	
Diabetes				0.415
No	3,247 (80.3)	3,005 (80.4)	242 (78.3)	
Yes	799 (19.7)	732 (19.6)	67 (21.7)	
CKD				
No	3,801(93.9)	3,504 (93.8)	297 (96.1)	0.123
Yes	245 (6.1)	233 (6.2)	12 (3.9)	
Heart disease				
No	3,487 (86.2)	3,222 (86.2)	265 (85.8)	0.890
Yes	559 (13.8)	515 (13.8)	44 (14.2)	
HDL (SD), mmol/L	1.3 (0.4)	1.3 (0.4)	1.4 (0.4)	<0.001
TC (SD), mmol/L	5.1 (1.0)	5.1 (1.0)	5.1 (1.1)	0.449
ASM (SD), kg	18.4 (3.5)	18.6 (3.4)	15.8 (3.1)	0.004
NHHR	3.3 (1.7)	3.3 (1.8)	3.0 (1.6)	<0.001
Quartiles of NHHR				0.001
Q1	1,002 (24.8)	889 (23.8)	113 (36.6)	
Q2	1,024 (25.3)	956 (25.6)	68 (22.0)	
Q3	1,004 (24.8)	934 (25.0)	70 (22.7)	
Q4	1,016 (25.1)	958 (25.6)	58 (18.8)	

CKD, chronic kidney disease; HDL, high-density lipoprotein cholesterol; TC, total cholesterol; ASM, appendicular skeletal muscle; NHHR, non-high-density lipoprotein cholesterol to high-density lipoprotein cholesterol ratio.

### 3.2 Association between NHHR and sarcopenia

After adjusting for possible confounders, a high NHHR was associated with a decreased likelihood of sarcopenia (Table 2). The association remained stable when NHHR was categorized into quartiles. Compared with the participants in quartile 1 (Q1:≤2.24), those in Q2 (2.25–3.03), Q3 (3.04–3.99), and Q4 (≥4.00) had adjusted ORs for sarcopenia of 0.51 (95% CI, 0.37–0.71; *p* < 0.001), 0.51 (95% CI, 0.37–0.71, *p* < 0.001), and 0.40 (95% CI, 0.28–0.58, *p* < 0.001), respectively.

**Table 2:** The relationship between NHHR and the risk of sarcopenia.

	Model 1	*p* value	Model 2	*p* value	Model 3	*p* value
Continuous NHHR	0.87 [0.80–0.95]	<0.01	0.83 [0.76–0.92]	<0.01	0.84 [0.76–0.92]	<0.001
Quartiles of NHHR						
Q1 (≤2.24)	1 (Ref)		1 (Ref)		1 (Ref)	
Q2 (2.25–3.03)	0.56 [0.41–0.77]	<0.001	0.49 [0.36–0.68]	<0.001	0.51 [0.37–0.71]	<0.001
Q3 (3.04–3.99)	0.59 [0.43–0.81]	0.001	0.49 [0.35–0.68]	<0.001	0.51 [0.37–0.71]	<0.001
Q4 (≥4.00)	0.48 [0.34–0.66]	<0.001	0.39 [0.28–0.56]	<0.001	0.40 [0.28–0.58]	<0.001
*p* for trend	<0.001		<0.001		<0.001	

Q, quartile; OR, odds ratio; CI, confidence interval; Ref, reference.

Model 1: crude model.

Model 2: adjusted for age, sex, education level, location, and marital status.

Model 3: adjusted for smoking status, drinking status, diabetes, hypertension, chronic heart disease, and kidney disease based on Model 2.

Furthermore, the association between NHHR and sarcopenia remained stable after PSM, indicating the robustness of our findings (Supplementary Table S2).

**Figure 2:**
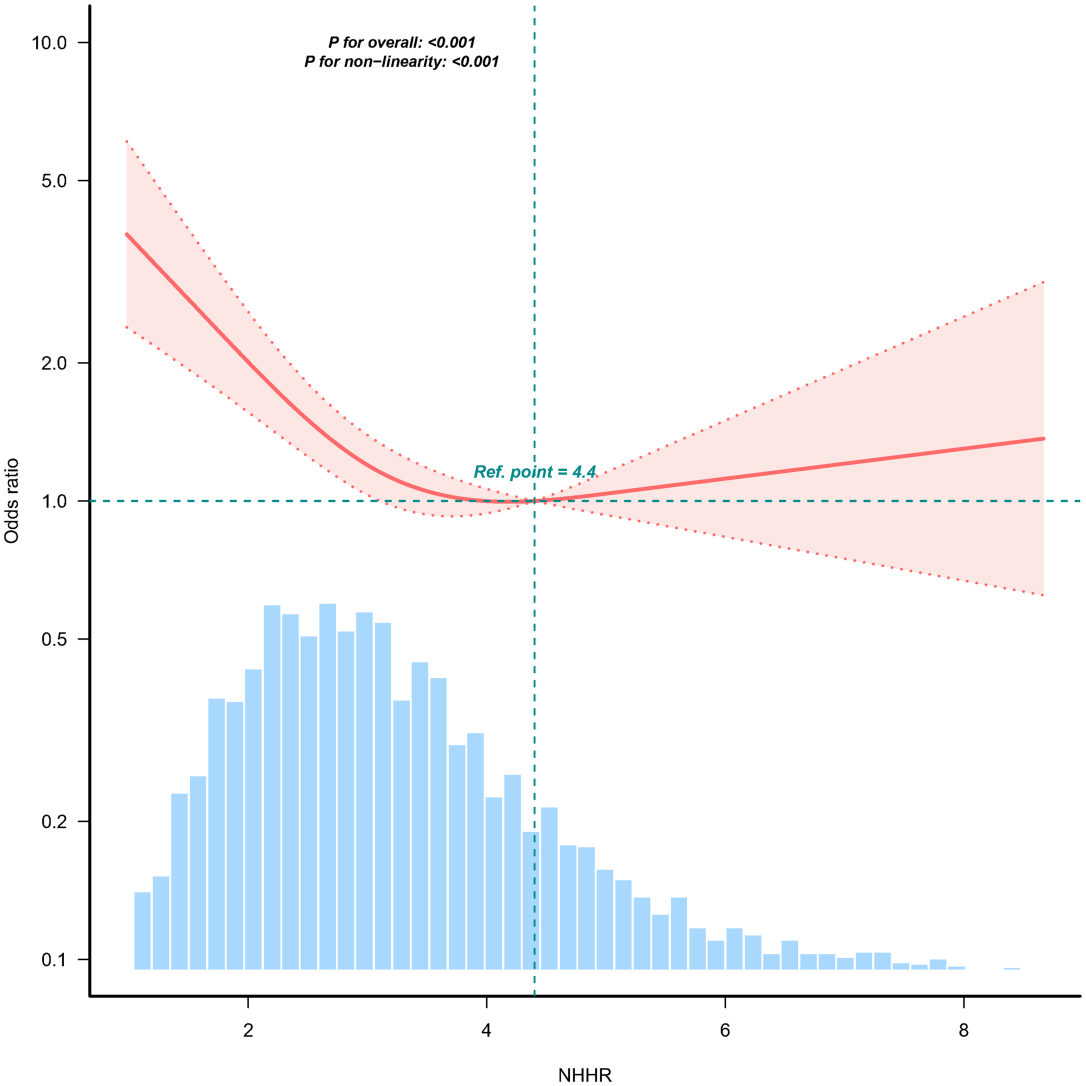
Association between the NHHR and the sarcopenia odds ratio. The solid and dashed lines indicate the predicted values and 95% confidence intervals, respectively. The restricted cubic spine model was adjusted for age, sex, education level, location, marital status, smoking status, drinking status, diabetes, hypertension, heart disease, and kidney disease. Only 98.5% of the data are shown.

The non–linear association between NHHR and sarcopenia was observed both before (Figure 2) and after (Supplementary Figure S1) PSM in the RCS. Before PSM, in the two-piecewise regression models, the OR for developing sarcopenia was 0.65 (95% CI, 0.55–0.78, *p* < 0.001) among the participants with an NHHR <4.4, whereas no significant association was found between NHHR and sarcopenia in those with an NHHR ≥4.4 (Table 3). Supplementary Figure S1 illustrates the results of the RCS analysis, demonstrating a stable non–linear trend between NHHR and sarcopenia regardless of sex matching (*p* for non-linearity <0.001).

**Table 3:** The relationship between NHHR and the risk of sarcopenia using two-piecewise regression models.

NHHR	Model 3	
	OR (95%CI)	*p-*value
<4.4	0.65 [0.55–0.78]	<0.001
≥4.4	1.14 [0.78–1.67]	0.50
Log-likelihood ratio test		0.001

NHHR, non-high-density lipoprotein cholesterol to high-density lipoprotein cholesterol ratio; OR, odds ratio; CI, confidence interval.

Model 3 was adjusted for age, sex, education level, location, marital status, smoking status, drinking status, diabetes, hypertension, heart disease, and kidney disease.

### 3.3 Stratified analyses

**Figure 3:**
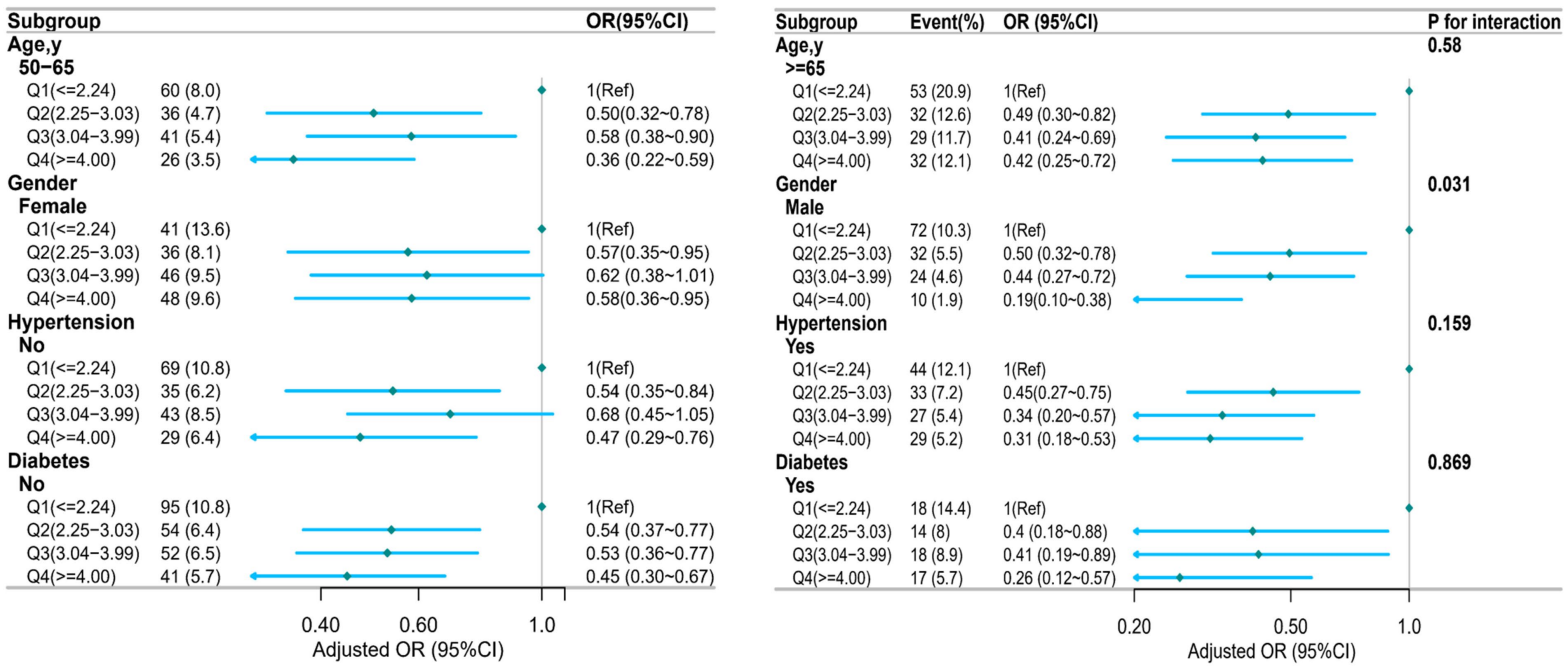
Subgroup analysis by age, sex, hypertension, and diabetes.

Analyses were stratified to evaluate possible modifications in the effect of NHHR on sarcopenia across different subgroups. There were no significant interactions in any of the subgroups after stratification by age, sex, hypertension, and diabetes (Figure 3). However, a statistically significant interaction was observed for sex (p for interaction = 0.031), indicating that the association between NHHR and sarcopenia may differ by sex. The stratified analysis after PSM showed that although higher NHHR levels (such as in the age group ≥65 years, and in the non-hypertension group) could reduce the risk of sarcopenia, the statistical significance was relatively weak. Overall, the heterogeneity of the association between NHHR and sarcopenia across different subgroups was minimal, suggesting that the impact of NHHR on sarcopenia risk was relatively consistent among various subgroups, consistent with the results before PSM (Supplementary Figure S2). After PSM, the interaction between sex and NHHR was no longer statistically significant (P for interaction >0.05), suggesting that the previously observed sex-specific heterogeneity may have been influenced by confounding factors or an imbalance in baseline characteristics.

### 3.4 Sensitivity analysis

Sensitivity analysis was performed to verify the robustness of the study findings. Individuals with extreme NHHR values (<0 or >6.61) were excluded, and 3,922 participants remained. The association between NHHR and the risk of sarcopenia remained consistent. Compared with the participants in the lowest quartile (Q1: ≤2.24), the adjusted ORs for NHHR in Q3 (3.04–3.99) and Q4 (≥4.00) were 0.52 (95% CI, 0.37–0.73, *p* < 0.001) and 0.39 (95% CI, 0.27–0.57, *p* < 0.001), respectively (Supplementary Table S1).

## 4 Discussion

Baseline data from CHARLS indicated that participants with sarcopenia were more likely to be older, female, and have higher HDL-C levels, and have lower NHHRs. Follow-up data revealed an inverse relationship between NHHR and sarcopenia risk. Each unit increase in NHHR was associated with a 16% reduction in sarcopenia risk (OR, 0.84; 95% CI, 0.76–0.92). Dose–response analysis further confirmed a nonlinear (L-shaped) association between NHHR and sarcopenia. Notably, the sex interaction that had been observed before matching disappeared after PSM, which may indicate that the initial interaction effect was partly attributable to residual confounding factors such as age and comorbidities. The consistency of the association after matching further supports the robustness of the relationship between NHHR and sarcopenia.

The results revealed a consistent and stable association between higher NHHR levels and reduced sarcopenia risk both before and after PSM. Before PSM, we identified a significant threshold at an NHHR of 4.4 in the nonlinear relationship with sarcopenia. Specifically, NHHR was negatively associated with the incidence of sarcopenia when it was lower than 4.4. Although NHHR has traditionally been considered a risk factor for cardiovascular diseases, our findings offer new insights into the potential positive impact of NHHR on sarcopenia in older adults. These results underscore the importance of screening for sarcopenia in older adults with very low NHHR levels in clinical practice.

Lipid profiles provide essential insights into lipid metabolism and overall health status and are commonly used to predict the risk of various diseases (26). Several studies have suggested a potential association between lipid profiles and sarcopenia. Notably, high HDL-C levels traditionally considered protective against cardiovascular disease have been linked to an increased risk of sarcopenia among middle-aged and older adults in China (15, 16). A study by Wang et al. (27) with 2,613 participants revealed that the TG/HDL-C ratio was inversely associated with sarcopenia (OR, 0.63; 95% CI, 0.49–0.81), which aligns with our findings. Similarly, Lin et al. (14) reported that the TG/HDL-C ratio inversely correlated with sarcopenia, noting that individuals in the highest quartile of TG/HDL-C had a lower risk of severe sarcopenia among patients with diabetes in China.

As an accessible and low-cost lipid marker, NHHR could serve as a useful tool in primary care settings, especially in low-resource environments where more expensive diagnostic tests may not be feasible. Its potential application in sarcopenia screening could offer a cost-effective alternative for early identification of participants who may be at risk for sarcopenia. Our findings revealed an inverse relationship between low NHHR and sarcopenia, consistent with recent epidemiological studies examining other health outcomes. Yu et al. (13) found that when NHHR values were below 2.72 and 2.83, each unit increase in NHHR corresponded to a 24% decrease in overall mortality and a 30% decrease in cardiovascular mortality. However, NHHR positively correlated with both overall and cardiovascular mortality when values exceeded the inflection point. Similarly, Liu et al. (28) reported a U-shaped relationship between NHHR and the incidence of major adverse cardiovascular and cerebrovascular events(MACCEs) in Chinese patients with coronary artery disease who underwent percutaneous coronary intervention (PCI), indicating that both low and high NHHR values were associated with an increased risk of MACCEs.

The findings of these studies highlight the importance of maintaining an appropriate NHHR, and our study also suggests that the lowest NHHR does not necessarily indicate the best health state. The possible reasons why lower NHHR values are linked to a higher risk of sarcopenia remain unclear. Excessively low NHHR values may result from high levels of HDL-C. Although HDL-C has traditionally been considered “good cholesterol” due to its protective effects against cardiovascular diseases and reduces all-cause mortality (29), recent studies have demonstrated that elevated HDL-C levels may paradoxically increase the risk of adverse cardiovascular outcomes and mortality (30). Differences in HDL particle composition between individuals with low and high HDL-C levels have been reported (31), which may potentially lead to altered HDL functions (32). Excessively elevated HDL-C levels have been shown to induce cellular senescence and become pro–inflammatory (33, 34). Increased levels of reactive oxygen species are induced, leading to the activation of the ubiquitin–proteasome cascade reaction, which subsequently promotes muscle proteolysis and results in reduced muscle mass (35, 36). Thus, the inverse association between NHHR and sarcopenia observed in our study may reflect complex interactions between lipid metabolism and inflammation. Further studies are warranted to elucidate these mechanisms.

In contrast to our findings, previous research has reported a positive association between NHHR and sarcopenia in American adults (17, 19). Our findings differ from those of previous studies, possibly due to variations in the study populations and the diagnostic criteria used for diagnosing sarcopenia. However, further studies are needed to clarify the mechanisms linking NHHR to sarcopenia risk.

Emerging evidence suggests that both lipid and glucose metabolism are influenced by genetic factors, contributing to atherosclerotic burden and metabolic fragility, which is particularly relevant in the aging population (37). These findings support the notion that composite lipid indexes, such as NHHR, may impact broader age-related outcomes such as sarcopenia. In addition, subclinical organ damage, especially kidney dysfunction, can often be detected at an early stage using sensitive biomarkers, even in the absence of overt clinical symptoms (38). These studies are relevant to understanding how metabolic imbalances may drive early changes in sarcopenia.

### 4.1 Strengths and limitations

As a nationwide and prospective cohort study, our research included a diverse representative sample. We employed three models to adjust for potential confounders, enhancing the robustness and validity of our findings. We also performed stratified analyses to explore this association across multiple subgroups.

However, this study has some limitations. First, although we employed multivariate logistic regression models, along with subgroup and sensitivity analyses, residual confounding effects cannot be entirely ruled out, particularly due to the self-reported nature of comorbidities and lifestyle factors. The reliance on self-reported data introduced potential biases such as misclassification or recall bias, which could have affected the accuracy of the estimates. Second, we exclusively recruited participants aged 50 years and above, so additional studies are needed to extend our findings to younger populations. Third, certain variables, such as chronic disease status, smoking, and alcohol consumption, were self-reported through questionnaires, which could have introduced recall bias. Fourth, this study used an equation to calculate muscle mass rather than direct techniques such as DXA. Although the equation has been validated and shows high agreement with DXA-measured ASM, potential misclassification exists, particularly in aging populations. Finally, as this study focused on Chinese older individuals, caution should be exercised when generalizing the findings to other populations.

## 5 Conclusion

In this study, we observed an inverse association between NHHR and sarcopenia. In older adults with a very low NHHR, screening for sarcopenia in clinical practice is crucial.

## Data Availability

The original contributions presented in the study are included in the article/Supplementary material, further inquiries can be directed to the corresponding author.

## Supplementary material

SUPPLEMENTARY FIGURE S1 Association between NHHR and the sarcopenia odds ratio after PSM. The solid and dashed lines indicate the predicted values and 95% confidence intervals, respectively. The restricted cubic spline model was adjusted for age, sex, education level, location, marital status, smoking status, drinking status, diabetes, hypertension, heart disease, and kidney disease. Only 98.5% of the data are shown.
