# Supplementary material for: Association between the non-high-density lipoprotein cholesterol to high-density lipoprotein cholesterol ratio and sarcopenia: evidence from CHARLS
Source: Front Public Health. 2025 Apr 30;13:1585986. doi: 10.3389/fpubh.2025.1585986 (PMC12074908; doi:10.3389/fpubh.2025.1585986)
Supplement: Supplementary file 1 [file Table_1.docx]

| Table S1 Characteristics of the participants in the original/matched set. | | | | | | | |
| --- | --- | --- | --- | --- | --- | --- | --- |
| Characteristic | Original data set | | |  | Matched Data set | | |
|  | Sex | | |  | Sex | | |
|  | Female | Male | SMD |  | Female | Male | SMD |
| N | 1732 | 2313 |  |  | 551 | 551 |  |
| Age (SD) | 59.5 (6.5) | 61.1 (7.1) | 0.236 |  | 61.0 (7.0) | 61.0 (7.2) | 0.003 |
| Education |  |  | 0.648 |  |  |  | 0.072 |
| No formal education | 1133 (65.4) | 804 (34.7) |  |  | 256 (46.5) | 274 (49.7) |  |
| Primary school | 298 (17.2) | 703 (30.4) |  |  | 154 (27.9) | 147 (26.7) |  |
| Middle school | 212 (12.2) | 528 (22.8) |  |  | 93 (16.9) | 89 (16.2) |  |
| High school or above | 89 ( 5.1) | 279 (12.1) |  |  | 48 ( 8.7) | 41 ( 7.4) |  |
| Married | 1516 (87.5) | 2121 (91.7) | 0.136 |  | 494 (89.7) | 484 (87.8) | 0.057 |
| Location in rural | 648 (37.4) | 741 (32.0) | 0.113 |  | 211 (38.3) | 202 (36.7) | 0.034 |
| Current smoker | 123 ( 7.1) | 1727 (74.6) | 1.89 |  | 123 (22.3) | 143 (26.0) | 0.085 |
| Current drinker | 237 (13.7) | 1551 (67.0) | 1.295 |  | 197 (35.8) | 196 (35.6) | 0.004 |
| Hypertension |  |  | 0.149 |  |  |  | 0.022 |
| No | 851 (49.1) | 1308 (52.4) |  |  | 283 (51.4) | 289 (52.5) |  |
| Yes | 881 (50.9) | 1005 (43.4) |  |  | 268 (48.6) | 262 (47.5) |  |
| Diabetes |  |  | 0.096 |  |  |  | 0.027 |
| No | 1352 (78.1) | 1894 (81.9) |  |  | 443 (80.4) | 437 (79.3) |  |
| Yes | 380 (21.9) | 419 (18.1) |  |  | 108 (19.6) | 114 (20.7) |  |
| CKD |  |  | 0.059 |  |  |  | 0.024 |
| No | 1641 (94.7) | 2159 (93.3) |  |  | 519 (94.2) | 522 (94.7) |  |
| Yes | 91 ( 5.3) | 154 ( 6.7) |  |  | 32 ( 5.8) | 29 ( 5.3) |  |
| Heart_disease |  |  | 0.118 |  |  |  | 0.032 |
| No | 1452 (83.8) | 2034 (87.9) |  |  | 475 (86.2) | 481 (87.3) |  |
| Yes | 280 (16.2) | 279 (12.1) |  |  | 76 (13.8) | 70 (12.7) |  |
| CKD: Chronic Kidney Disease | | | | | | | |
