# Supplementary material for: Association between the non-high-density lipoprotein cholesterol to high-density lipoprotein cholesterol ratio and sarcopenia: evidence from CHARLS
Source: Front Public Health. 2025 Apr 30;13:1585986. doi: 10.3389/fpubh.2025.1585986 (PMC12074908; doi:10.3389/fpubh.2025.1585986)
Supplement: Supplementary file 2 [file Table_2.docx]

| Table S2. The relationship between NHHR and the risk of sarcopenia in the matched set. | | | | | | |
| --- | --- | --- | --- | --- | --- | --- |
|  | Model 1 | p value | Model 2 | p value | Model 3 | p value |
| Continuous NHHR | 0.89 [0.76–1.03] | 0.103 | 0.84 [0.71–0.99] | 0.041 | 0.84 [0.71–0.99] | 0.042 |
| Quartiles of NHHR | | | | | | |
| Q1 (≤2.34) | 1 (Ref) |  | 1 (Ref) |  | 1 (Ref) |  |
| Q2 (2.35–3.13) | 0.56 [0.33–0.96] | 0.036 | 0.52 [0.29–0.92] | 0.024 | 0.52 [0.29–0.92] | 0.026 |
| Q3 (3.14–4.10) | 0.49 [0.28–0.85] | 0.012 | 0.42 [0.23–0.76] | 0.005 | 0.42 [0.23–0.77] | 0.005 |
| Q4 (≥4.11) | 0.46 [0.26–0.81] | 0.007 | 0.38 [0.20–0.70] | 0.002 | 0.38 [0.20–0.88] | 0.002 |
| p for trend | 0.004 | | 0.001 | | 0.001 | |
| Q, quartiles; OR, odds ratio; CI, confidence interval; Ref, reference.  Model 1: crude model.  Model 2: adjusted for age, sex, education level, location, and marital status.  Model 3: adjusted for smoking status, drinking status, diabetes, hypertension, chronic heart disease, and kidney disease based on Model 2. | | | | | | |
