# Supplementary figures and images for: Association between the non-high-density lipoprotein cholesterol to high-density lipoprotein cholesterol ratio and sarcopenia: evidence from CHARLS
Source: Front Public Health. 2025 Apr 30;13:1585986. doi: 10.3389/fpubh.2025.1585986 (PMC12074908; doi:10.3389/fpubh.2025.1585986)

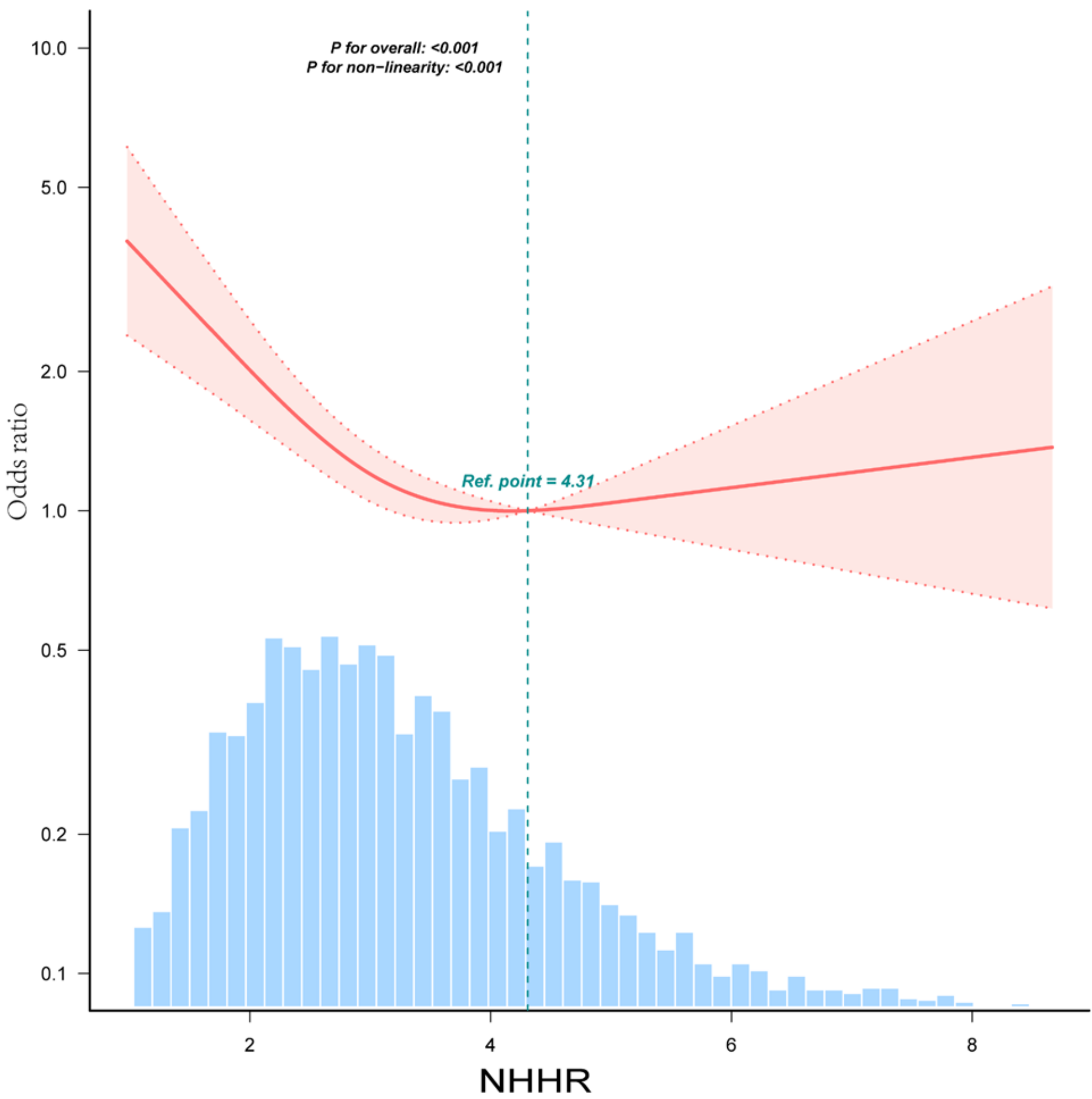

Supplement: SUPPLEMENTARY FIGURE S1 — Association between NHHR and the sarcopenia odds ratio after PSM. The solid and dashed lines indicate the predicted values and 95% confidence intervals, respectively. The restricted cubic spline model was adjusted for age, sex, education level, location, marital status, smoking status, drinking status, diabetes, hypertension, heart disease, and kidney disease. Only 98.5% of the data are shown. [file Data_Sheet_1.pdf]

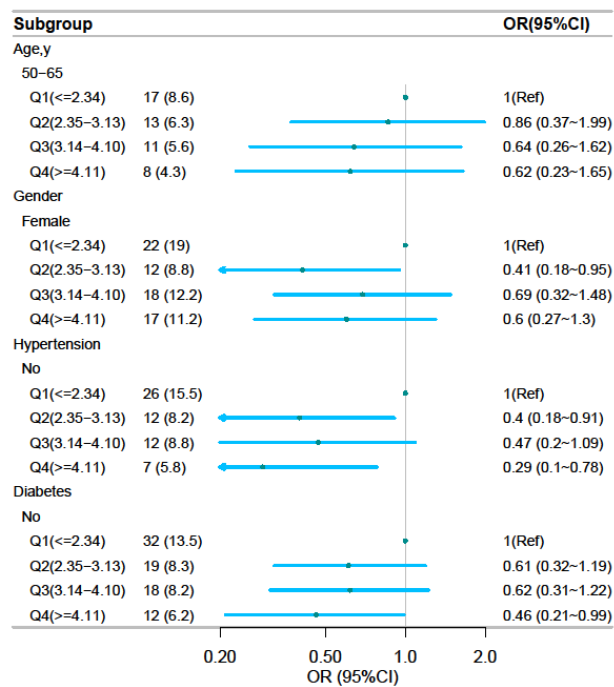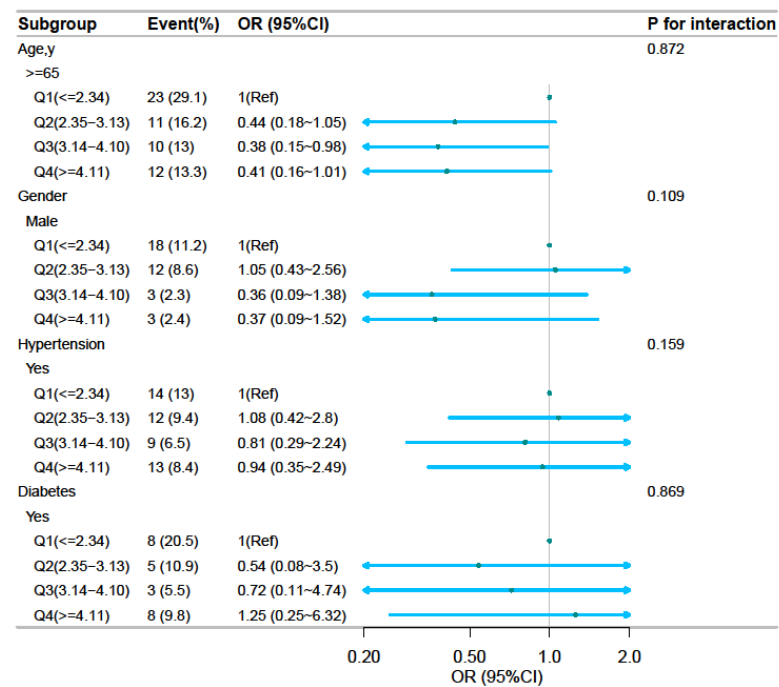

Figure S2

Subgroup analysis by age, sex, hypertension, and diabetes after PSM.

Supplement: Supplementary file 4 [file Data_Sheet_2.pdf]
